# Measuring and interpreting platelet-leukocyte aggregates

**DOI:** 10.1080/09537104.2018.1430358

**Published:** 2018-02-20

**Authors:** Michaela Finsterbusch, Waltraud C. Schrottmaier, Julia B. Kral-Pointner, Manuel Salzmann, Alice Assinger

**Affiliations:** Department for Vascular Biology and Thrombosis Research, Centre for Physiology and Pharmacology, Medical University of Vienna, Vienna, Austria

**Keywords:** Platelet-leukocyte aggregates, inflammation, flow cytometry, live cell imaging

## Abstract

Platelets, besides their specialised role in haemostasis and atherothrombosis, actively modulate innate and adaptive immune responses with crucial roles in immune surveillance, inflammation and host defence during infection. An important prerequisite for platelet-mediated changes of immune functions involves direct engagement with different types of leukocytes. Indeed, increased platelet-leukocyte aggregates (PLAs) within the circulation and/or locally at the site of inflammation represent markers of many thrombo-inflammatory diseases, such as cardiovascular diseases, acute lung injury, renal and cerebral inflammation. Therefore, measurement of PLAs could provide an attractive and easily accessible prognostic and/or diagnostic tool for many diseases. To measure PLAs in different (patho-)physiological settings in human and animal models flow cytometric and microscopic approaches have been applied. These techniques represent complementary tools to study different aspects relating to the involvement of leukocyte subtypes and molecules, as well as location of PLAs within tissues, dynamics of their interactions and/or dynamic changes in leukocyte and platelet behaviour. This review summarises various approaches to measure and interpret PLAs and discusses potential experimental factors influencing platelet binding to leukocytes. Furthermore, we summarise insights gained from studies regarding the underlying mechanism of platelet-leukocyte interactions and discuss implications of these interactions in health and disease.

## Introduction

Platelets are small anucleate cells with crucial function in primary haemostasis, where they are essential to seal injured vessels via thrombi formation at sites of vascular injury. Due to their high sensitivity to thrombotic and immunologic factors, platelets further represent susceptible sentinels that are ready to launch immediate and potent immune responses. Platelet activation and concomitant degranulation enables platelets to engage with leukocytes via soluble factors and physical interaction facilitated by a variety of receptors (1). Most importantly receptors involved in direct interactions include platelet P-selectin (CD62P) and CD40 ligand (CD40L), as well as PSGL-1, CD40 and Mac-1 (integrin α_M_β_2_, CD11b/CD18) on leukocytes (2–4). Platelet-leukocyte interactions are further stabilised by crosstalk of numerous additional receptor/ligand pairs that were reviewed previously (1,5) and trigger mutual activation and release of granular content by both platelets and leukocytes, thereby modulating leukocyte function and fine-tuning immune responses (5). Importantly, platelet-leukocyte interactions facilitate leukocyte recruitment and extravasation to sites of inflammation (6,7), promote leukocyte release of pro-inflammatory mediators (8,9), oxidative burst, phagocytosis, release of neutrophil extracellular traps (NETs) (10,11), and may also dampen inflammation under some pathological conditions (12–14).

**Figure 1.**
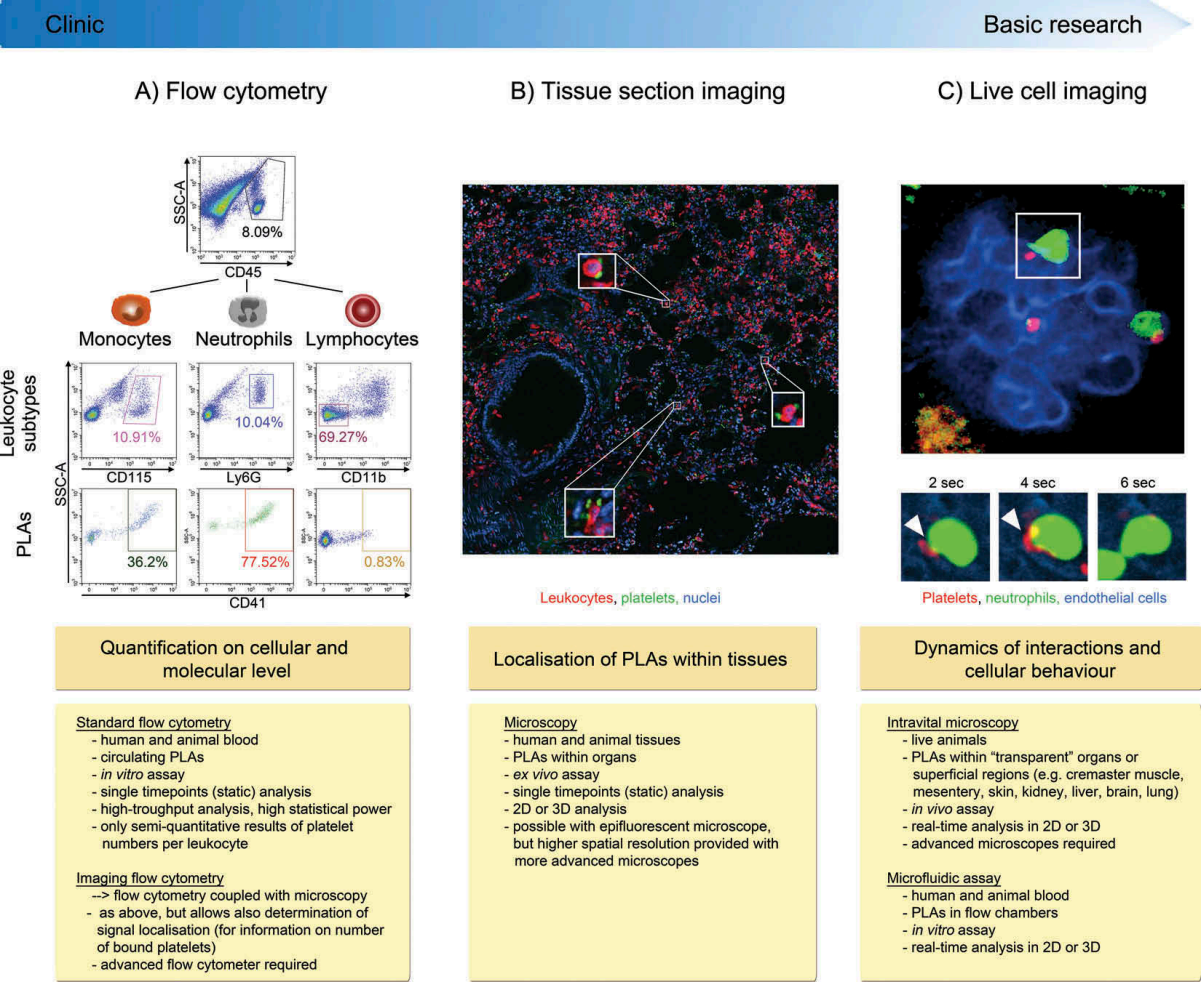
Methods to investigate platelet-leukocyte aggregate (PLA) formation. (A) Flow cytometry represents a high-throughput approach to quantify circulating PLAs in human and animal blood and to investigate expression of involved molecules. Due to its fast protocol and independence of highly specific machines, standard flow cytometry is readily usable in clinical settings. Panels show gating strategy to determine platelet hetero-aggregates with distinct leukocyte subtypes in stimulated murine blood. CD45 (pan-leukocyte marker), Ly6G (neutrophil marker), CD11b (marker to exclude cells other than lymphocytes) and CD41 (pan-platelet marker). (B) Tissue sectioning adds information about location of PLAs within human or animal tissues in conjunction with quantitative and qualitative evaluation of PLAs. However, preparation and evaluation of samples is more time-consuming and does not allow for high-throughput analysis, making tissue sectioning less attractive for clinical laboratories. Images show PLAs in inflamed murine lung histology sections (neutrophils labelled with anti-CD45 and platelets with anti-CD41). C) Concomitant live analysis of multiple cell types in animals *in vivo* or of human or animal blood cells in microfluidic devices provides information about dynamic changes of platelet-leukocyte interactions and their effect on cellular effector functions within physiologic microenvironments or under semi-physiological conditions under flow. Live cell imaging to investigate PLAs requires state-of-the-art microscopy facilities and areas of interest that are accessible for microscopy, thus making it well suited for basic research but not for clinical applications. Panel shows multiphoton image sequences of a platelet (labelled with anti-CD49b) interacting with a GFP^+^ neutrophil within glomerular capillaries of a LysM-GFP mouse over time (endothelial cells labelled with anti-CD31). PLA: platelet-leukocyte aggregate, SSC-A: side scatter––area, DAPI: 4′,6-Diamidin-2-phenylindol.

Extensive flow cytometric analysis and the development of advanced microscopy techniques have considerably extended our knowledge of platelet-leukocyte interactions and their implication in health and disease. Elevated PLA levels are associated with a range of acute and chronic thrombo-inflammatory conditions such as cardiovascular diseases (CVD), acute lung injury (ALI), rheumatoid arthritis, inflammatory bowel disease, stroke, and glomerulonephritis (15–22). Hence, PLAs could become an attractive parameter for diagnostic and/or prognostic analysis. However, variable results particularly in relation to PLA levels for both basal and inflammatory conditions between different laboratories have been reported, which might be due to different methods used to analyse PLAs, sample handling, and/or different species used. This review gives an overview on approaches used to investigate PLAs (Figure 1) and highlights factors influencing the results. Furthermore, we summarise recent findings on the role of PLAs in a variety of diseases and underline the potential and advantages of advanced flow cytometry and microscopy – in particular live cell imaging tools – for future studies.

## Translational aspects of animal models

Animal and in particular mouse models are invaluable to understand the role of PLAs under steady-state and inflammatory conditions. However, there are distinct differences between humans and mice that have to be taken into consideration. In healthy humans for example, neutrophils represent the dominant leukocyte subpopulation with about 40%–70%, followed by lymphocytes (20%–45%) and monocytes (2%–10%). In contrast, mice exhibit up to 84% lymphocytes, 11% neutrophils and 2% monocytes (23) and levels may vary considerably depending on strain, age and housing conditions. Furthermore, due to different affinities of platelet P-selectin to PSGL-1 on distinct leukocytes, platelets preferentially bind to monocytes followed by neutrophils and to a lesser extent to lymphocytes (24,25). This may explain the discrepancy in basal PLA formation between species: i.e. 10%–20% in human (26), and 1%–10% in mice (27–29). Furthermore, differences exist in receptor expression, with the most prominent being protease activated- and Fcγ receptors (30), hence also mechanisms of platelet activation might differ between mouse and human. Notably, however, the main receptors facilitating PLAs (i.e. P-selectin, CD40L, GPIbα, GPVI, GPIIb/IIIa on platelets and PSGL-1, CD40, Mac-1 on leukocytes) are consistently involved in both mice and human (2,4,6,8,31–38).

## Methods to measure PLAs

### Sample preparation

PLA formation, in particular when involving monocytes, is extremely sensitive to environmental factors. Therefore, blood drawing (e.g. type of anticoagulant) and sample preparation (e.g. temperature, centrifugation, erythrocyte lysis and storage) represent critical steps to obtain comparable results (26). For example, venepuncture leads to lower basal platelet-monocyte aggregates (PMAs) compared to intravenous cannulae (39). Furthermore, anticoagulation by EDTA, followed by sodium citrate, prevents artificial PMAs more efficiently than heparin, hirudin and PPACK (39,40). Storage time and temperature of blood samples also influence plasmatic cytokine levels (41) and microvesicle release (42), that in turn can severely affect platelet activation and artificial platelet-leukocyte binding. To guarantee consistent PLA levels up to 24 hours, appropriate fixation and storage at 4°C is important (39,40,43).

### Measurement of circulating PLAs

Flow cytometry is the method of choice to measure circulating PLAs of both human and animal origin, representing a quick and highly sensitive technique that enables concomitant analysis of platelet activation and PLA formation within the same sample (44). Furthermore, this approach allows rapid recording and analysis of thousands of leukocytes per specimen, thereby reducing inherent sample variations and facilitating the evaluation of rare leukocyte subpopulations.

Flow cytometry is based on fluorescence-labelling of platelets and leukocytes in whole blood. For basic PLA analysis, only two fluorescently labelled antibodies (a platelet-specific and a pan-leukocyte-specific antibody) are required (25,26). Characteristic side scatter properties of leukocyte subtypes already permit basic discrimination of platelets binding to neutrophils, monocytes and lymphocytes (25). However, leukocyte subtype-specific antibodies generate more accurate data: e.g. antibodies against CD66b (human) or Ly6G (mouse) to label neutrophils, CD14 and CD16 or CD64 (human), or CD115 and Ly6C (mouse) for monocyte subsets, CD3, CD4 and CD8 (mouse and human) for T-cells, CD19 (human) or CD19 and B220 (mouse) to label B-cells, and CD56 (human) or NK1.1 (mouse) for NK-cells (25,45–48).

Following sample recording, PLAs are quantified as the percentage of total leukocytes that also stain for a platelet-specific marker (Figure 1A). Thus, PLA values are independent of absolute leukocyte numbers in patient blood and can be evaluated also in leukopenic patients. Similarly, due to the abundance of platelets over leukocytes in the circulation, PLAs can also be measured during thrombocytopenia. Increased circulating PLAs have been associated with thrombocytopenia in dengue fever (49). However, it remains unknown whether PLA levels are affected by haematologic changes, such as altered platelet count or haematocrit themselves, or if thrombocytopenia is caused by elevated platelet activation, sequestration and PLA formation

A limitation of standard flow cytometry is that absolute numbers of bound platelets are not directly assessed and potential platelet-derived microvesicles fused to leukocytes could influence results. Mean or median fluorescence intensity (MFI) of platelets bound to leukocytes can be used for relative comparison and semi-quantitative analysis (24). Recent technological advances linking flow cytometry with fluorescence microscopy, so called “imaging flow cytometry”, combine advantages of both methods for PLA measurement: high-throughput analysis with concomitant determination of signal localisation, enabling direct assessment of both number of bound platelets as well as involved interaction molecules and coincidental events (50,51).

### Microscopy techniques to measure static and dynamic PLAs within tissues

Histochemical and immunofluorescent imaging of frozen or paraffin-embedded tissue sections have provided valuable information about the location of platelet recruitment and PLAs within tissue microenvironments of numerous organs (22,52–55). These techniques, in particular when coupled with advanced confocal or electron microscopy, provide high resolving images to visualise platelet-leukocyte interactions at locations even within opaque tissues of both human and animal origin (Figure 1B). However, microscopy of tissue sections can only display static end-point analysis and do not resemble the full complexity of the dynamic behaviour of cells. The development of *in vivo* imaging techniques, such as intravital microscopy (IVM) further allows to track cells in live animals over longer time periods in organs including cremaster muscle, liver, lung, brain and kidney (Figure 1C) (56,57), and have been greatly beneficial in analysing dynamic interaction between platelets and leukocytes (e.g. duration of interactions and behavioural changes in leukocytes and/or platelets upon contact) (10,53,55,58–61).

Live cell imaging largely relies on fluorescent tools, including probes, antibodies, biosensors or reporter mice for visualising cells and/or structures without interfering with their phenotype and function. Early epifluorescence IVM studies primarily utilised intravascular administration of nonspecific cell dyes (e.g. rhodamine 6G), or *ex vivo* labelling and adoptive transfer of platelets into donor animals (58,62–64). However, the lack of clear distinctions between specific cell types using unspecific dyes limits the analysis of PLAs. Also, *ex vivo* staining and transfusion of purified platelets does not reflect the *in vivo* situation as it does not allow for analysis of unlabelled endogenous platelets and further harbours the risk of platelet activation due to *in vitro* handling.

Alternatively, more direct approaches to visualise PLAs *in vivo* include intravenous administration of fluorochrome-conjugated monoclonal antibodies. In this context, several markers specifically label platelets, including CD41 (GPIIb) (60), CD42 (GPIb) (61) and CD49b (α2 integrin subunit) (10,59,60,65). While the use of antibodies against these proteins enables clear identification of platelets interacting with other cells, some antibodies affect platelet function under certain conditions. For example, high dose of anti-CD41 monoclonal antibody affects platelet aggregation and inflammatory responses in mice (66), and proplatelet formation of megakaryocytes *in vitro* (67). Alternatively, reporter mouse lines expressing fluorescent proteins in platelets (55), or platelet subsets (65) can be used for visualising PLA dynamics in different organs.

Simple epifluorescent microscopy coupled with brightfield transillumination has been applied to track platelet-leukocyte interactions over time in some settings (58,61,68). However, this approach is very limited as it offers relatively low spatial and temporal resolution of processes on a cellular level. State-of-the-art imaging platforms to study platelet events include multiphoton and confocal microscopy, featuring markedly increased tissue penetration and three dimensional imaging. This is particularly beneficial for the analysis of platelet behaviour within thicker tissues and a high dynamic range (e.g. within structures such as glomeruli in the kidney, where vessels are hardly aligned in a single plane). Furthermore, to capture rapid platelet interactions which occur in liver (59) or kidney microvasculature (Finsterbusch et al., unpublished data) high frame rate imaging, as achieved e.g. via spinning disc microscopy, is essential.

### *In vitro* techniques to measure PLA dynamics

Microfluidic assays represent another microscopy-based approach to study platelet-leukocyte interactions in real time (11,69–74). Typically, isolated cells or whole blood are perfused through a chamber coated with immobilised proteins or cells, allowing precise control of soluble and cellular input. High or low physiological shear rates can be applied using pumps mimicking conditions observed in different blood vessels. Platelets and leukocytes, similar to IVM, can be visualised by cell tracker or specific antibodies, and careful sample handling is required to avoid cell activation.

While being less physiological than *in vivo* imaging, microfluidic assays represent a more cost-effective method to analyse platelet-leukocyte interaction dynamics under well-defined conditions and moreover allows the investigation of human cells. Various modifications in microfluidic devices exist including parallel plate flow chambers (75), cone and plate assay (76), Stamper-Woodruff assay (77) and a mouse-driven *ex vivo* flow chamber (78), allowing high-throughput analysis and/or analysis under defined haemodynamics that mimic specific (patho-)physiological conditions.

## Platelet-leukocyte interactions in pathologic conditions

**Figure 2.**
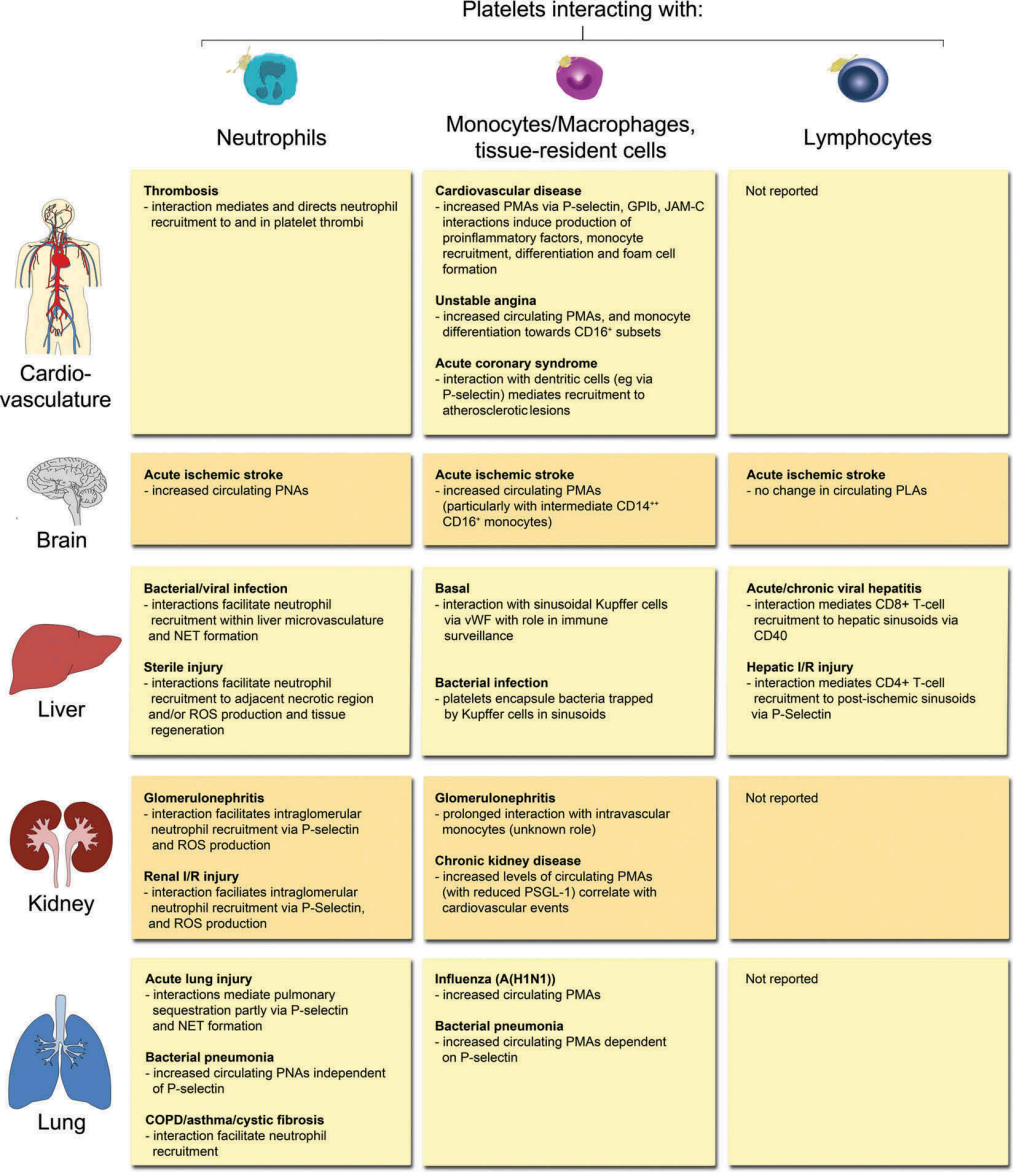
Overview of PLA formation and their effects in (thrombo-) inflammatory pathologies of selected organs. Physical interactions of platelets with neutrophils, monocytes/macrophages, tissue-resident leukocytes or lymphocytes have been reported in a number of (thrombo-)inflammatory diseases of the circulatory system, brain, liver, kidney, and lung. Effects of platelet-leukocyte binding on cellular and/or molecular responses are summarised for individual leukocyte subtypes. COPD: chronic obstructive pulmonary disease, I/R: ischemia/reperfusion, JAM-C: Junctional adhesion molecule-C, NET: neutrophil extracellular trap, PMA: platelet-monocyte aggregate, PNA: platelet-neutrophil aggregate, PSGL-1: P-selectin glycoprotein ligand 1, ROS: reactive oxygen species, vWF: von Willebrand factor.

Emerging studies suggest associations between PLAs and pro-inflammatory effects in numerous pathological conditions, including CVD, rheumatoid arthritis, inflammatory bowel disease and autoimmune cerebral inflammation, which were previously reviewed (1,79,80). In this review we focus on platelet-leukocyte interactions in acute ischemic stroke, renal diseases, and hepatic as well as lung inflammation and infection, and give an overview on the leukocyte types involved (Figure 2).

### Acute ischemic stroke

Circulating levels of PLAs, significantly increase within 24 hours after acute ischemic stroke and represent a more sensitive marker for platelet activation compared to P-selectin and platelet aggregation (81). In particular, increased binding of platelets to monocytes and neutrophils were observed (82–84), while only few reports of platelet-lymphocyte interactions exist (85). An increase in intermediate CD14^++^ CD16^+^ monocytes and their binding to platelets 7 days post percutaneous coronary intervention after myocardial infarction is further associated with an increased risk for ischemic stroke besides other adverse cardiovascular events (86). Of note, one study reported that changes in PLA levels occur mainly in large and small vessel disease, but not in cardioembolic disease (87).

In a murine model of cerebral artery occlusion, IVM studies showed platelet-leukocyte interactions within post-ischemic venules (88). These events might be facilitated via GPIIb/IIIa or P- and L-selectin, as inhibition of these molecules in an experimental model of ischemic stroke and reperfusion reduces the increase in PLAs (89). In patients suffering from acute ischemic stroke, increased PLAs at the early phase correlate with neurological deterioration (82,90), which can be partly prevented by pretreatment with statin and aspirin (90). Intake of aspirin also significantly reduces PMA levels in stroke-recovering patients (91) and anti-platelet therapy is beneficial in the prevention of secondary stroke after cardiovascular events. Furthermore, in patients with large-artery atherosclerosis-induced stroke aspirin and clopidogrel together reduce platelet aggregation and PLA formation more efficiently than aspirin alone (92). These results indicate that inhibiting several platelet activation pathways might be beneficial in patients by limiting ischemic stroke recurrence and neurologic deterioration.

### Liver diseases

Chronic liver disease and cirrhosis are often associated with thrombocytopenia, which also correlates with disease outcome. Thrombocytopenia is caused by various processes including increased breakdown and pooling of platelets in the spleen (93,94), decreased platelet production (95) and also local accumulation of platelets in the liver (96), where they can interact with leukocytes and contribute to tissue injury and fibrosis. Furthermore, the extent of platelet accumulation in donor liver allografts was reported to correlate with organ function in liver transplant patients (97). Indeed, numerous experimental models demonstrated a role for platelet-leukocyte interactions in several (patho-)physiological conditions of the liver, including immune surveillance, homeostasis, liver regeneration, microbial sensing and host defence against invading pathogens.

Under steady-state conditions, platelets transiently interact with Kupffer cells (KCs) in liver sinusoids of mice via von Willebrand factor, thereby scanning the liver microvasculature for signs of pathogen invasion (59). This immune surveillance might be particularly important in the liver, given the close connection to the gastrointestinal tract and thus, being one of the first lines of host defence. Indeed, during bacterial infection, platelet-KC interactions increase in duration and result in platelet aggregates encapsulating bacteria previously trapped by KCs (59). Furthermore, TLR4-mediated platelet activation leads to profound binding of platelets to neutrophils in the liver (11), an event critical for promoting NET formation and hence bacterial elimination (10). Large platelet-neutrophil aggregates (PNAs) are formed also during viral infection within the liver microvasculature and precede NET release (98). This suggests that platelets modulate leukocyte activation and NET release via direct interaction to a variety of pathogens.

In the inflamed liver platelet interaction with leukocytes further facilitates leukocyte recruitment and adhesion. In a model of sterile liver inflammation, following induction of a focal region of injury, platelets immediately pave the sinusoidal endothelium adjacent to the necrotic lesion (99), which allows for neutrophil crawling along immobilised platelets towards the injured site supporting tissue repair. Similarly, platelet aggregates occur along hepatic sinusoids in necro-inflammatory foci in mice with acute viral hepatitis. This is substantial for recruitment of virus-specific CD8^+^ T cells causing hepatocellular injury (53,54) potentially via CD40L (100).

Platelets are further involved in both acute and chronic viral hepatitis as shown by attenuated accumulation of CD8^+^ T cells and virus-nonspecific inflammatory cells into the liver upon anti-platelet therapy (using aspirin and clopidogrel) (101,102). Similarly, platelet inhibition in clinically relevant sterile liver inflammation, such as ischemia/reperfusion injury (as observed following transplantation) reduces liver damage via blocking platelet- and P-selectin-dependent CD4^+^, but not CD8^+^ T cell recruitment to post-ischemic sinusoids (58). Nicotinamide adenine dinucleotide phosphate oxidase 2 might also play a role in mediating platelet-neutrophil interactions in this setting (61). In another sterile injury model, following bile duct ligation-induced cholestasis, platelet aggregates support leukocyte recruitment to liver sinusoids and postsinusoidal venules involving P-selectin (63). Hence, these studies emphasise a mechanistic link between platelet-leukocyte interactions and leukocyte recruitment followed by tissue damage in a wide range of inflammatory liver disease.

### Renal diseases

While many renal pathologies are caused by leukocyte-mediated tissue injury (103,104), clinical and experimental data suggest a central role for platelets in a number of chronic and acute kidney disorders. In experimental mouse models and patients with different types of glomerulonephritis platelets accumulate in the kidneys and/or platelet-derived factors are systemically elevated (21,22,105–108). Accordingly, some clinical studies (using only a small patient cohort, however) show beneficial effects of anti-platelet therapy in glomerulonephritis patients by reducing circulating platelet aggregates, albuminuria and/or glomerular filtration rate (109–113) and anti-platelet drugs are indeed commonly prescribed in Japanese hospitals to patients with IgA nephropathy (114). The contribution of platelets in the induction of renal inflammation via direct interactions with leukocytes is further supported by the requirement of platelet P-selectin for neutrophil recruitment and/or neutrophil-mediated injury in experimental acute postischemic renal failure (115) and glomerulonephritis (21,22). Neutrophil-depletion on the other hand prevented platelet accumulation within glomerular capillaries in a model of immune-complex-induced glomerulonephritis (22), emphasising the importance of the interplay between platelets and neutrophils during this disease. Indeed, our unpublished data suggest constitutive on/off interactions between platelets and neutrophils or monocytes in glomerular capillaries (Finsterbusch et al. unpublished), leukocyte subsets that both were reported to contribute to renal injury in acute glomerular inflammation (116,117).

Moreover, PLAs occur in blood of patients with systemic lupus erythematosus (106), a systemic autoimmune disease, which commonly results in renal diseases. Platelet interaction with plasmacytoid dendritic cells via CD40L/CD40 potentiates IFN-α secretion *in vitro*, and anti-platelet therapy protects from kidney failure in lupus-prone mice. Increased platelet counts are also found in renal transplants (118) and PLAs contribute to the high cardiovascular mortality rate in renal transplant, dialysis and chronic kidney disease patients (119–121), highlighting PLAs as a potential biomarker for renal diseases and prognosis of patients.

### Lung inflammation and infection

Platelets are important for pulmonary function by maintaining vascular integrity of the alveolar-capillary structure, as severe thrombocytopenia (approximately 1% residual platelets) in mice leads to alveolar haemorrhages and decreased survival during transfusion-related acute lung injury (TRALI). However, even low platelet levels (approx. 6%–10% residual platelets) are sufficient to prevent haemorrhagic complications, indicating a low threshold platelet level to maintain basic haemostatic platelet function (122). In response to local and systemic triggers, however, platelets can also facilitate leukocyte recruitment towards sites of injury and thereby support inflammatory responses and defence mechanisms against invading pathogens. Acid-induced ALI for example leads to rapid P-selectin-dependent formation of PNAs and deficiency of platelet P-selectin abrogated pulmonary neutrophil sequestration, thereby ameliorating oedema formation and facilitating gas exchange (15). Similarly, in LPS-induced ALI, TRALI or systemic LPS/zymosan A-induced ALI platelet depletion by up to 90%, thus not falling below threshold levels for maintaining basic haemostasis, dampens neutrophil recruitment into the alveolar compartment, tissue injury and vascular leakage (15,123–125). However, in contrast to acid-induced ALI, platelet-mediated effects on neutrophil recruitment are independent of P-selectin in LPS-induced ALI, but still require PSGL-1 (123,125). These studies indicate that, depending on the underlying pathology, PNAs are not always mediated by classic P-selectin/PSGL-1 binding, but rather by other receptor-ligand interactions or via soluble mediators. Indeed, in LPS-induced ALI platelet-derived CXCL4 and CCL5 control neutrophil infiltration via induction of CXCL2-release by alveolar macrophages (126,127). Furthermore, sequestrated platelets in the pulmonary microcirculation of TRALI-induced mice promote NET formation (128).

While platelet-assisted leukocyte infiltration and activation may cause undue tissue injury in the absence of live pathogens, platelet-leukocyte interactions are essential in infectious lung diseases to support bacterial clearance. In this context, thrombocytopenia increases bacterial burden in some inflammatory settings, such as *Streptococcus pneumoniae*-induced pneumonia while not affecting neutrophil infiltration (129). Furthermore, P-selectin-deficiency results in reduced circulating PMAs during *Klebsiella pneumoniae*-induced pneumonia, but has no effect on pulmonary leukocyte sequestration (130). However, thrombin inhibition reduces PNA formation *in vitro* and exacerbates sepsis in *K. pnemoniae*-induced pneumosepsis, but fails to reduce neutrophil recruitment or NET formation *in vivo* (131). This indicates that platelet-leukocyte interactions during infectious ALI are highly complex and multifaceted and further research is necessary to unravel the underlying mechanism of platelet-mediated immune modulation in lung diseases.

## Concluding remarks

Advanced microscopic and flow cytometric approaches have provided evidence that platelets directly interact with leukocytes and have helped to gain key insights into the role and consequences of PLA formation in health and disease. Since numerous inflammatory diseases are associated with increased PLAs, platelet-binding to different subtypes of leukocytes might be used as a potential biomarker for the assessment of both risk factors and disease progression. Flow cytometry, in particular when combined with readily available kits (132), represents an easy and fast approach to determine PLA formation in whole blood that could also be applied to the clinics. However, to allow for validation of PLAs as biomarker in the future, protocols for standardised sample preparation and robust reference values have to be established first. Further research is warranted to understand the underlying mechanism of PLA formation and to unravel the effect of platelet-leukocyte interplay as well as their potential as therapeutic target in various diseases.
